# Timing of infestation influences virulence and parasite success in a dynamic multi-host–parasite interaction between the invasive parasite, *Philornis downsi*, and Darwin’s finches

**DOI:** 10.1007/s00442-020-04807-5

**Published:** 2020-12-01

**Authors:** Arno Cimadom, Sabine Tebbich

**Affiliations:** Department of Behavioural Biology, University of Vienna, Althanstrasse 14, 1090 Vienna, Austria

**Keywords:** Host–parasite interaction, Virulence, *Philornis downsi*, Darwin’s finches, Infestation pattern, Host preference

## Abstract

Recently commenced host–parasite interactions provide an excellent opportunity to study co-evolutionary processes. Multi-host systems are especially informative because variation in virulence between hosts and temporal changes provides insight into evolutionary dynamics. However, empirical data under natural conditions are scarce. In the present study, we investigated the interaction between Darwin’s finches and the invasive fly *Philornis downsi* whose larvae feed on the blood of nestlings. Recently, however, the fly has changed its behavior and now also attacks incubating females. Two sympatric hosts are affected differently by the parasite and parasite load has changed over time. Our study observed a reversal of trends described two decades ago: while, currently, small tree finches (*Camarhynchus parvulus*) experience significantly higher parasite load than warbler finches (*Certhidea olivacea*), this was the opposite two decades ago. Currently, fledging success is higher in warbler finches compared to small tree finches. Our data indicate that not only intensity but also timing of infestation influences hosts’ reproductive success and parasite fitness. During incubation, prevalence was higher in warbler finches, but once chicks had hatched, prevalence was 100% in both species and parasite load was higher in small tree finches. Furthermore, our results suggest faster development and higher reproductive success of *P. downsi* in small tree finch nests. A change in host preference driven by larvae competition could have led to the reversal in parasite load.

**Electronic supplementary material:**

The online version of this article (10.1007/s00442-020-04807-5) contains supplementary material, which is available to authorized users.

## Introduction

Parasites cause direct or indirect fitness loss by impairing reproduction and survival (Clayton and Moore 1997; Lehmann 1993; Loye and Zuk 1991; Price 1980; Tschirren et al. 2009) and, hence, they represent a major selective force. Virulence refers to the degree of fitness loss caused by pathogens and parasites. It is the result of complex interactions between host traits (resistance and tolerance) and parasite traits (exposure and pathogenicity; Johnson et al. 2012; Råberg 2014; Råberg et al. 2009). Thus, virulence can vary greatly between and within-host species (Bull 1994), which in turn influences if and how an evolutionarily stable equilibrium between hosts and parasites is reached (Rigaud et al. 2010). Multi-host systems are especially informative because variation in virulence between hosts and changes in virulence over time provide an excellent opportunity to observe the evolutionary dynamics of host–parasite interactions. However, empirical data gathered under natural conditions that show the interplay between tolerance, resistance, virulence and parasite productivity in multi-host systems are scarce (Manzoli et al. 2018). Recently established host–parasite interactions (e.g., due to an invasion by a new parasite species) allow the tracking of adaptative processes in hosts and parasites from the outset.

An example of one recently established, dynamically changing multi-host model field system is the interaction between the invasive ectoparasitic fly *Philornis downsi* and different species of Darwin’s finches, which are highly affected hosts of this fly on the Galápagos Islands. This obligate bird parasite was introduced to the Galápagos archipelago in the 1960s (Causton et al. 2006; Kleindorfer and Sulloway 2016). Free-living adult flies deposit their eggs in bird’s nests where their hematophagous larvae hatch (Fessl et al. 2006b). The first instar larvae usually develop in the chicks’ nostrils, whereas the second and third instar larvae live in the bottom layer of the nest and suck blood from the nestlings (Dudaniec and Kleindorfer 2006; Fessl et al. 2006b; O’Connor et al. 2010a), causing severe fitness loss in their avian hosts (reviewed in Fessl et al. 2017).

However, the level of virulence of *P. downsi* differs between host species on the Galápagos Islands. Galápagos mockingbirds (*Mimus parvulus*) tolerate the introduced parasite well, while sympatric medium ground finches (*Geospiza fortis*) suffer high brood loss due to parasitism (Knutie et al. 2016). Similarly, our recent study showed that in the same habitat, warbler finches (*Certhidea olivacea*) are less affected by *P. downsi* than closely related small tree finches (*Camarhynchus parvulus)* (Cimadom et al. 2019).

The aim of the present study was to investigate the causes of the differences in *P. downsi* virulence between the warbler finch and the small tree finch. More specifically, we wanted to investigate how parasite intensity in different phases of the hosts’ breeding cycle influences host reproductive success and parasite developmental success.

High parasite intensity is one source of elevated virulence (Stearns and Koella 2008) but it is also important to consider the number of parasites in relation to host body mass (Knutie et al. 2016). Cimadom et al. (2019) showed that in an average nest, the parasite burden increased with chick mass: a 50% higher burden was found in small tree finches (~ 13 g) than in warbler finches (~ 8 g). The timing of infestation within the breeding cycle may also influence virulence. There are strong indications of a recent change in parasite infestation behavior on Santa Cruz Island. Previously, larvae were only found in nests once chicks had hatched, but since 2012, we have observed *P. downsi* larvae of all larval stages in nests during the incubation stage when no chicks were present (Cimadom et al. 2016). At this stage, it is likely that the larvae are feeding on incubating females (Huber et al. 2010; Koop et al. 2013). For newly hatched chicks, a small difference in total parasite intensity might lead to significant differences in virulence (Arendt 1985; Kleindorfer and Dudaniec 2016). Furthermore, as *P. downsi* likely increases its blood consumption with increasing larval size and larval stage, not only the total amount of larvae but also the size and/or type of instar present when chicks hatch might have a significant effect on the virulence of *P. downsi*.

For the parasite’s reproductive success, the developmental stage of the larvae when the host resource availability terminates (either dead chicks or fledged hosts) is also a relevant factor as it determines its fitness. Parasite fitness depends on how fast the larvae develop (which depends on host quality) and on the mean duration of host availability (which depends on virulence). It has been suggested that the change in infestation behavior shown by *P. downsi* might be a result of intraspecific competition between the parasites: due to increasing parasite intensities in hosts over the last two decades, hosts die at an earlier age which reduces the time during which resources are available for developing *P. downsi* larvae (Kleindorfer and Dudaniec 2016; Kleindorfer et al. 2014). The time window of host resource availability determines not only parasite pupation success but also pupae size and ultimately adult fecundity (Honek 1993; Kleindorfer et al. 2014; Quiroga and Reboreda 2013; Spalding et al. 2002). In newly established multi-host systems, parasite developmental speed and, thus, host suitability might differ between host species, which could lead to the evolution of host preferences.

In the present study, we investigated the dynamics of a multi-host system under natural conditions by analyzing longitudinal data on virulence, infestation patterns and parasite developmental success in two sympatric host species, the warbler finch and small tree finch. Specifically, we addressed the following questions: Are there differences between the two hosts in the intensity and timing of infestation within the breeding cycle? Does this affect virulence and/or developmental success of the parasite? Are there changes in *P. downsi* intensity between and within hosts over time?

## Methods

### Study site and nest monitoring

The study was conducted at the “Los Gemelos” site in the humid highland of Santa Cruz Island, Galápagos (S 00°37′20″–45″ W 90°23′00″–15″, 500–600 m a.s.l.) between 2012 and 2017 (except for 2013). The study site is one of the last remnants (ca. 100 ha) of native *Scalesia pedunculata* forest on Santa Cruz (Mauchamp and Atkinson 2011).

We monitored the nests of warbler finches and small tree finches over five breeding seasons from 2012 to 2017 (January–March 2012, January–April 2014, January–May 2015, January–April 2016 and January–April 2017). Nest monitoring followed the procedure described in Cimadom et al. (2014). Once a nest was determined to be in the incubating or feeding stage, a pole-mounted endoscopic camera (Findoo 3.6, dnt Innovation, 26789 Leer, Germany) was used to assess the number of eggs or nestlings present and to determine whether the nestlings were alive. Monitoring intervals were adjusted to the status of the nest: three-day intervals were used to monitor for nests during incubation, two-day intervals for nests with chicks. Nests were monitored daily around the presumed fledging date of chicks.

After breeding failure or successful fledging, all monitored nests were collected in separate, sealed plastic bags and dismantled in the laboratory on the same day to count the number of *P. downsi* larvae, pupae and empty puparia. Larvae were categorized as either Small (< 0.5 mm), Medium (5–10 mm) or Big (> 10 mm). Parasite intensity for a nest was defined as the total number of *P. downsi* individuals in the nest at the termination of breeding activity. Since it has been found that the intensity per chick decreases with increasing brood size—an indication of a parasite dilution effect (Dudaniec et al. 2007), the number of chicks could also have been relevant for this study. However, here we did not evaluate parasite intensity per chick because (1) clutch size did not differ between the two species (warbler finch: 2.28 ± 0.62, mean ± SD, *N *= 113; small tree finch: 2.38 ± 0.76, *N *= 78; Mann–Whitney test: *U *= 4798.5, *p *= 0.25), (2) the maximum number of chicks was not known for all nests and (3) the number of chicks changed over the nestling period, as chicks often die sequentially. The age of chicks at the termination of breeding activity was recorded for each nest as described in Cimadom et al. (2014). Chicks of both species fledge at about 14 days of age (Cimadom et al. 2014). Because of the onset of the breeding seasons differed between years, we calculated a standardized start of incubation for each nest as (DIS − minDIS)/(maxDIS − minDIS), where DIS is the date of incubation start for a given nest, minDIS is the earliest recorded date of incubation start and maxDIS is the latest recorded date of incubation start in each year (values range from 0 to 1).

### Size of eclosed flies

To test for differences in the size of eclosed *P. downsi* flies, we measured the head width (eye to eye) to the nearest 0.1 mm with a caliper. *P. downsi* flies emerged from 26 small tree finch nests and 31 warbler finch nests in 2012.

### Statistical analysis

All statistical analyses were performed using the program R, version 3.5.1 (R Core Team 2018). For all models described below, analysis-of-variance tables (type-II) were calculated using the *Anova* procedure in the *car* package (Fox and Weisberg 2011).

#### *Philornis downsi* prevalence and intensity

We used separate general linear models (GLMs; binomial family and logit link function) to test for the effect of host species, standardized start of incubation and year on *P. downsi* prevalence (*P. downsi* present or absent in nest) in nests that failed during the incubation phase (*N *= 109) and in nests with chicks (*N *= 528).

We compared parasite prevalence between abandoned nests and nests that failed for other reasons during incubation using Chi^2^-tests for each host species separately, pooling the data from all five years.

We used a GLM (quasi-Poisson error structure) to test for the effect of host species and year on *P. downsi* intensity in infested nests that failed during the incubation phase (*N *= 70).

Effects of host species, age of chicks at nest failure or fledging (linear and quadratic), the interaction term host species * age of chicks, and year on parasite intensity (number of *P. downsi* individuals per nest) were evaluated using a GLM with a negative-binomial error structure (because of overdispersion). For this analysis, only nests with chicks were used (*N *= 513).

#### Nesting outcome and chick age

We used GLMs (quasi-Poisson error structure) to test for differences between the two Darwin’s finch species and years (as co-variable) in chick age at the end of nesting activity (failure or fledged, *N *= 513) and in chick age at death (failed nests only, *N *= 315).

In a previous study, we found a higher nest failure rate in the small tree finch than in the warbler finch (Cimadom et al. 2019). Here, we used the same data to see if the previously described breeding failure was related to chick age. We did this using a GLM (Poisson distribution) where the number of failed nests was a dependent variable and the age of chicks, host species and the interaction between host species * age of chicks as independent variables. The total number of nests per host species and the year was defined as an offset variable, to calculate probability values for each chick age, host species and year combination.

#### Developmental success of the parasite

*Philornis downsi* undergoes three larval stages before pupation (Fessl et al. 2006b): the first, second and third instar stages. Only third instar larvae can pupate, so upon occurrence of host death, it is only larvae that have reached the third instar stage which can pupate and subsequently emerge as flies (Kleindorfer et al. 2014; Knutie et al. 2016). Thus, the total number of third instar larvae and of pupae provides a good estimate of the parasite’s developmental success. *Philornis downsi* eggs take at least 4–7 days to develop into pupae (Kleindorfer et al. 2014). This means that the age that chicks reach in the nest (which may vary between host species) is linked to parasite developmental success.

Big larvae are always third instar larvae. Medium larvae include both second and third instar larvae and Small larvae are either first or second instars. In 2012, all larvae (all three size categories together) from each nest were kept in separate Petri dishes (diameter 90 mm) that were lined with a small, piece of moistened paper towel. Pupae from the nests were stored in gauze-covered plastic cups (for each nest separately). Larvae and pupae were kept at 27 °C and 65% relative humidity until eclosion or death. In total, out of 724 Big and 633 Medium larvae collected, 913 pupated. If we assume that all Big larvae pupated, we can conclude that 29.9% of the medium larvae were also third instars that managed to pupate. We conservatively defined mature *P. downsi* larvae, which are no longer dependent on a living host, as the number of Big larvae found per nest. As a proxy for parasite developmental success, we used the total number of mature *P. downsi* larvae and pupae per nest. To test if the number of mature larvae and pupae differed between the two host species, we ran a GLM (quasi-Poisson error structure) with the total number of mature *P. downsi* larvae and pupae as the dependent variable, host species as the independent variable and year as a co-variable (*N *= 513).

The proportion of mature larvae and pupae per nest was used as an indicator of pupation success (following Kleindorfer et al. 2014). A detailed analysis of the proportion of mature larvae, evaluating the effects of host species, age of chicks at failure or fledging (linear and quadratic), year and the interaction of host species * age of chicks on the proportion of mature larvae and pupae within a nest was conducted by constructing a GLM with a quasi-binomial error structure and logit link function (*N *= 513).

In a further step, we focused on nests with young chicks (≤ 5 days old, *N *= 160), since for young chicks, a small difference in total parasite intensity might lead to significant differences in virulence (Arendt 1985; Kleindorfer and Dudaniec 2016). We tested to see whether there were differences between the host species in total parasite intensity (GLM with quasi-Poisson error structure), pupation success (GLM with quasi-binomial error structure) and the number of mature (big larvae and pupae) and immature (small and medium) larvae separately (GLMs with quasi-Poisson error structure). In all these models, the year was included as a co-variable.

To test whether the size of eclosed flies differed between the host species, we ran a linear mixed model (LMM) with fly eye distance as the dependent variable and host species, age of chicks at failure or fledging and sex of fly as fixed factors and nest provenance as a random factor (random intercept) using the package l*me4* (Bates et al. 2015). The sex ratio of eclosed flies did not differ between the two host species and did not change over the breeding season (Causton et al. 2019) and was, therefore, not included in the analysis.

#### Long-term shifts in parasite intensity

To test for changes in *P. downsi* intensity over the last decade, we compared pooled data from 1998 to 2005 (Dudaniec et al. 2007) with our present data from 2012 to 2017 for the warbler finch and the small tree finch separately using one-sampled t-Tests.

## Results

### *Philornis downsi* prevalence and intensity during the incubation phase

**Fig. 1:**
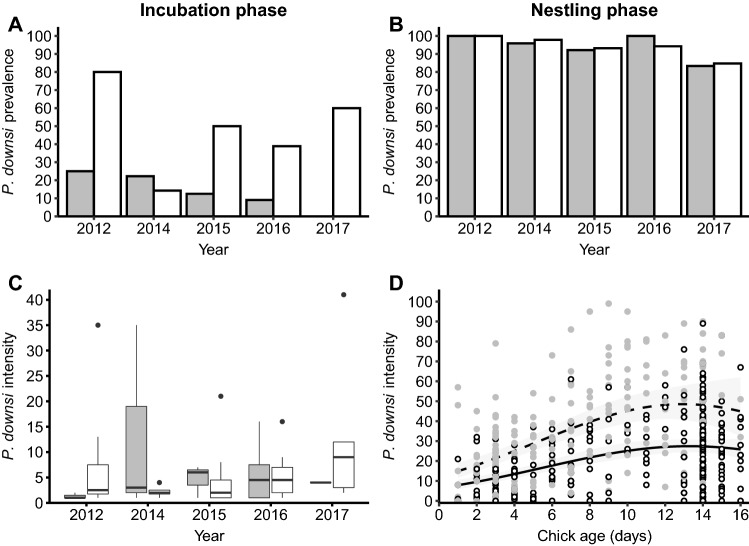
*Philornis downsi* infestation in the incubation (left column) and nestling phase (right column). Difference in *P. downsi* prevalence (percentage of infested nests) in **a** incubating nests and **b** nests with chicks of the small tree finch (gray) and the warbler finch (white) from 2012 to 2017. **c** *P. downsi* intensity in infested small tree finch (gray) and warbler finch (white) nests in the incubation phase from 2012 to 2017. Boundaries of the boxes represent 1st and 3rd quartiles, the black line within the boxes marks the median and whiskers extend from the median to the largest and lowest value within 1.5*IQR (interquartile range). **d** Predicted *P. downsi* intensity (± 95% CI) in warbler finch nests (solid line) and small tree finch nests (dashed line) in the nestling phase in relation to chick age. Fitted lines indicate the model’s predicted values for each set of observed values of the independent variables. Raw data are plotted as dots (small tree finch in gray and warbler finch in white)

*Philornis downsi* prevalence in nests that failed during incubation was significantly higher in warbler finch nests than in small tree finch nests (*X*^2^ = 16.62, df = 1, *p *≪ 0.001, Fig. 1a, Table S1) and differed among years (*X*^2^ = 9.47, df = 4, *p *= 0.05, Fig. 1a, Table S1). Parasite prevalence in small tree finch nests that were being incubated ranged from 0 to 25% and from 14.3 to 80% in warbler finch nests being incubated—it was highest in 2012 in both species. Parasite prevalence in nests being incubated did not change within the breeding season (*Χ*^2^ = 0.08, df = 1, *p *= 0.77, Table S1).

There was no significant difference in *P. downsi* intensity in infested incubation phase nests between the two host species (X^2^ = 0.004, df = 1, *p *= 0.95, Fig. 1c, Table S2) nor did it differ among study years (X^2^ = 4.43, df = 4, *p *= 0.35, Fig. 1c, Table S2). Median *P. downsi* intensity in both infested incubation phase warbler finch and small tree finch nests was 2.5 (warbler finch: range = 1-41, *N *= 52; small tree finch: range = 1-35, *N *= 18).

### Prevalence and *Philornis downsi* intensity during the nestling phase

Over all five study years, *P. downsi* prevalence during the nestling phase did not differ between the two host species (*X*^2^ = 0.03, df = 1, *p *= 0.85, Fig. 1b, Table S1) but varied among years (*X*^2^ = 27.86, df = 4, *p *≪ 0.001, Fig. 1b, Table S1). Prevalence in small tree finch nests ranged from 83% to 100% and from 85% to 100% in warbler finch nests and it was lowest in 2017 in both species (Table S3).

Analysis of *P. downsi* intensity per nest revealed that the parasite load per nest differed significantly between species (*X*^2^ = 55.1, df = 1, *p *≪ 0.001, Fig. 1d, Table S2) with warbler finch nests containing fewer parasites (22.8 ± 1.0 parasites per nest, *N *= 290) than small tree finch nests (mean ± SE parasites per nest: 34.4 ± 1.6, *N *= 223). Furthermore, the parasite load of both species increased with chick age until Day 11 and then leveled off from Days 11 to 15 (linear *X*^2^ = 23.4, df = 1, *p *≪ 0.001; and quadratic *X*^2^ = 8.9, df = 1, *p *= 0.003, Fig. 1d, Table S2). This relationship did not differ significantly between the two host species (host species*chick age: *X*^2^ = 0.14, df = 1, *p *= 0.70, Table S2). The parasite load differed among years (*X*^2^ = 13.0, df = 4, *p *= 0.011, Table S2) with 2017 nests exhibiting the lowest numbers of *P. downsi* (Table S3).

### Nesting outcome and chick age

The age of warbler finch chicks at the end of nest activity (failure or fledging) was significantly greater than that of small tree finch chicks (*X*^2^ = 57.8, df = 1, *p *≪ 0.001, Table S3 and S4) and differed among years (*X*^2^ = 21.0, df = 4, *p *= 0.001, Table S4). In both species, chick age was greatest in 2017 (mean ± SE age of warbler finches: 12.5 ± 0.46 Days; mean ± SE age of small tree finches: 8.8 ± 0.80 Days, Table S3). When data were pooled for all five study years, mean chick age was 10.7 ± 0.28 (mean ± SE) in the warbler finch and 7.4 ± 0.29 in the small tree finch. However, mean age at chick death did not differ between species (*X*^2^ = 0.62, df = 1, *p *= 0.43, Table S4) or among years (*X*^2^ = 3.37, df = 4, *p *= 0.50, Table S4). When data were pooled for all five study years, age at chick death was 6.3 ± 0.3 (mean ± SE) in the warbler finch and 6.0 ± 0.3 in the small tree finch (Table S3).

**Fig. 2:**
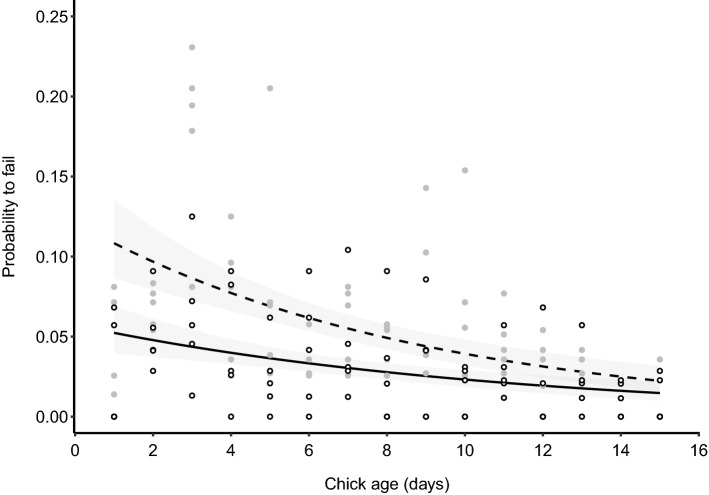
Predicted probability of failure (± 95% CI) of warbler finch (solid line) and small tree finch nests (dashed line) in relation to chick age. Fitted lines indicate the model’s predicted values for each set of observed values of the independent variables. Raw data are plotted as dots (small tree finch in gray and warbler finch in white)

The probability of failure was lower for warbler finch nests compared to small tree finch nests (*X*^2^ = 60.7, *p *< 0.001, Fig. 2, Table S5) and decreased in both finch species with increasing age of the chicks (*X*^2^ = 29.9, *p *< 0.001, Fig. 2, Table S5). This relationship did not differ between the two species (host species*chick age: *X*^2^ = 0.65, *p *= 0.42, Table S5).

### Developmental success of the parasite

The number of mature larvae and pupae in nests with chicks was significantly higher in the small tree finch than the warbler finch (*X*^2^ = 8.70, df = 1, *p *= 0.003, Table S6) and did not differ significantly among years (*X*^2^ = 1.31, df = 4, *p *= 0.86, Table S6). Over all five study years, the mean number of mature larvae and pupae was 20.6 ± 1.4 (mean ± SE) per nest in small tree finch nests and 15.8 ± 0.8 per nest in warbler finch nests.

**Fig. 3:**
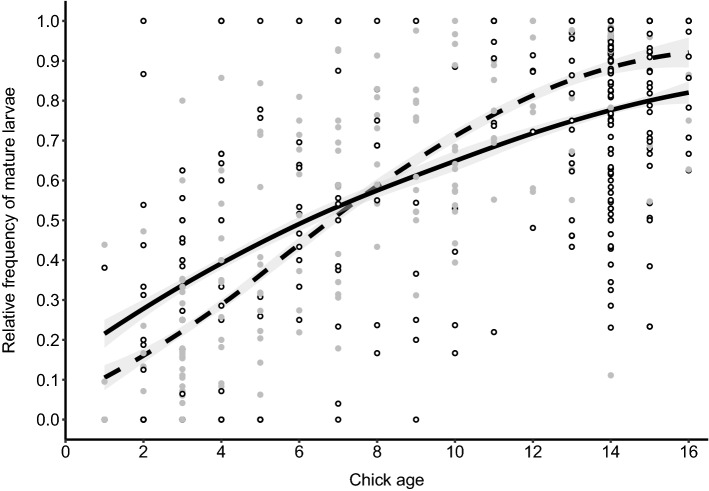
Predicted relative frequency of mature *P. downsi* larvae (± 95% CI) in nests of the warbler finch (solid line) and the small tree finch (dashed line) in relation to the age of host chicks. Fitted lines indicate the model’s predicted values for each set of observed values of the independent variables. Raw data are plotted as dots (small tree finch in gray and warbler finch in white)

The proportion of mature larvae and pupae increased significantly with chick age (linear: *X*^2^ = 48.28, df = 1, *p *≪ 0.001; quadratic: *X*^2^ = 1.78, df = 1, *p *= 0.18, Table S6). This increase, however, was different in the two Darwin’s finch species (host species*chick age: *X*^2^ = 21.94, df = 1, *p *≪ 0.001, Table S6). In warbler finch nests, the proportion of mature *P. downsi* larvae and pupae was higher for nests with young chicks but increased more slowly and reached lower levels in nests with older chicks compared to small tree finch nests (Fig. 3). Furthermore, the proportion of mature larvae and pupae differed among years (*X*^2^ = 43.20, df = 4, *p *≪ 0.001, Table S6): it was lowest in 2012 and 2016 and highest in 2014 and 2017 (Table S3).

Analysis of the early nestling phase (inclusion only of nests with ≤ 5-day-old chicks) confirmed lower parasite intensity (*X*^2^ = 23.47, df = 1, *p *≪ 0.001, Table S7) and a higher proportion of mature larvae and pupae (*X*^2^ = 11.53, df = 1, *p *= 0.001, Table S7) in warbler finch nests compared to small tree finch nests. The number of mature larvae and pupae in nests with ≤ 5-day-old chicks did not differ between the two host species (*X*^2^ = 0.18, df = 1, *p *= 0.67, Table S7), but small tree finch nests with young chicks contained more immature (Small and Medium) larvae than warbler finch nests (*X*^2^ = 32.23, df = 1, *p *≪ 0.001, Table S7).

**Fig. 4:**
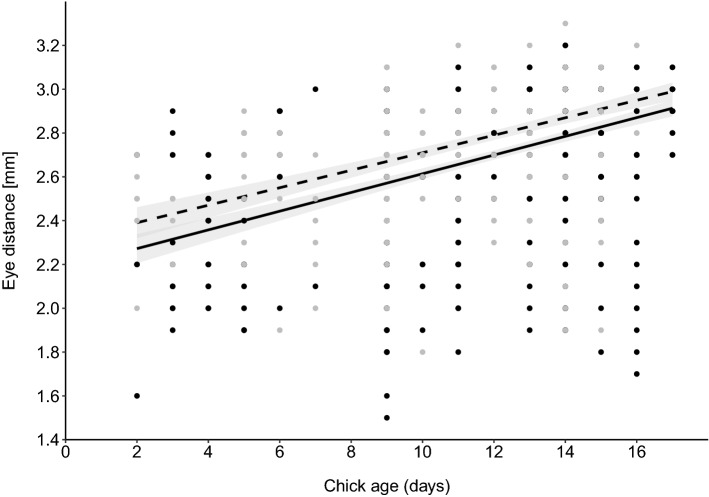
Predicted eye distance (± 95% CI) of female (solid line) and male (dashed line) *P. downsi* flies in relation to the age of host chicks. Fitted lines indicate the model’s predicted values for each set of observed values of the independent variables. Raw data are plotted as dots (small tree finch in gray and warbler finch in white)

Mean size of eclosed *P. downsi* flies did not differ between host species (mean ± SE eye distance for warbler finch nests was 2.72 ± 0.20 mm and 2.55 ± 0.23 mm for small tree finch nests; *X*^2^ = 0.04, df = 1, *p *= 0.84, Table S8). However, size increased with chick age (*X*^2^ = 61.08, df = 1, *p *≪ 0.01, Fig. 4, Table S8) and male flies were significantly bigger than female flies (*X*^2^ = 40.31, df = 1, *p *≪ 0.001, Fig. 4, Table S8).

### Long-term shifts in parasite intensity

**Fig. 5:**
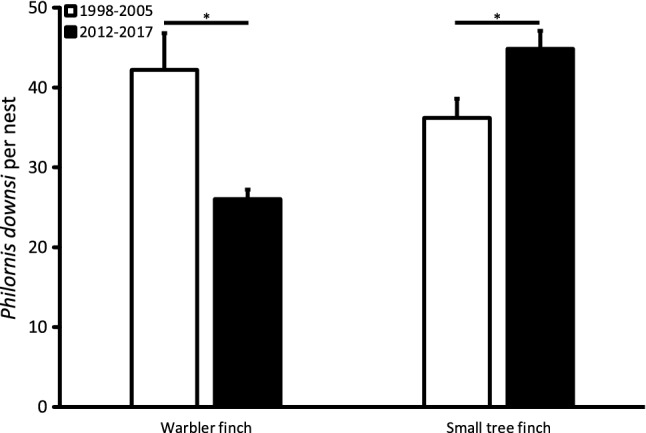
Number of *P. downsi* (mean ± SE) in warbler finch and small tree finch nests with nestlings ≥ 6 days old in the years 1998–2005 (Dudaniec et al. 2007) and 2012–2017

Compared to pooled data from 1998 to 2005 (Dudaniec et al. 2007), *P. downsi* intensity in nests with chicks ≥ 6 days has decreased in the warbler finch (one-sample *t* Test: N_1998-2005_ = 21, N_2012-2017_ = 228, t = 3.4, *p *= 0.002, Fig. 3) and increased in the small tree finch (one-sample *t*-Test: *N*_1998–2005_ = 33, *N*_2012–2017_ = 125, t = 2.6, *p *= 0.011, Fig. 5).

## Discussion

Our study showed that infestation patterns, as manifested by parasite prevalence and intensity, differed between the warbler finch and the small tree finch. Furthermore, these patterns changed within the breeding cycle suggesting a difference in the timing of infestation between the two hosts. During the incubation phase, *P. downsi* was more prevalent in the warbler finch than in the small tree finch and parasite intensity did not differ in infested incubation phase nests between the two hosts. However, once chicks hatched, prevalence was over 80% in both species, but small tree finch nests had higher parasite numbers than warbler finch nests over the whole nestling phase.

Differences in parasite intensity between the two hosts could be caused by differences in host attractiveness and/or host suitability. Currently, it is not clear how female flies find their hosts but olfactory cues are likely to be involved (Cha et al. 2016). Since small tree finch chicks are bigger than warbler finch chicks, they probably produce more olfactory cues which may make it easier for flies to detect them. Host suitability, on the other hand, may depend on factors such as nest characteristics (Kleindorfer and Dudaniec 2009; Sage et al. 2018) and host body mass (Kleindorfer and Dudaniec 2009). The fact that small tree finches have larger nests and larger chicks could explain why parasite intensity is higher than in warbler finch nests once chicks have hatched. However, this hypothesis is not in line with the finding of greater parasite intensity in warbler finches compared to small tree finches between 1998 and 2005.

One explanation for higher parasite prevalence in warbler finch nests during incubation could be that in some years, the breeding onset of warbler finches is earlier than of small tree finches. In these years, their incubation phase nests are the first or only available food source for the parasite at the beginning of the breeding season. However, parasite prevalence in nests being incubated did not change within the breeding season (standardized start of incubation) in either of the two hosts. Alternatively, the pattern could be a consequence of the overall higher density of warbler finch nests. Warbler finches are approximately 1.6 times more common than small tree finches (Cimadom et al. 2014, unpublished data) and it is possible that flies using only visual cues will encounter them more easily. The cause of higher prevalence of *P. downsi* in warbler finch nests during the incubation phase remains unclear. Detailed studies on fly behavior with cameras at the nest could provide relevant data such as when and how often adult flies visit the nests during the incubation and nestlings’ phase. Additionally, genetic studies like those presented in Dudaniec et al. (2010) could reveal the number of different female flies and the number of eggs they oviposited in each nest over the entire host’s breeding cycle.

### Implications for parasite virulence

Virulence may depend not only on the parasite load relative to chick mass and mechanisms of tolerance (e.g., Knutie et al. 2016) but also on the age at which the hosts are exposed to the parasite. The magnitude of the difference in virulence between the two host species is higher in young chicks. In nests with young chicks, the abundance of mature *P. downsi* larvae is the same in both species but small tree finch nests contain more immature larvae. This latter difference is likely to be the cause of the increased virulence in young small tree finch chicks. More specifically, first instar larvae develop predominantly in the nares (or ear cavities) of chicks (Fessl et al. 2006b), where they cause tissue damage and beak malformation (Galligan and Kleindorfer 2009), which could contribute to the negative fitness effects of the parasite during this larval stage.

### Implications for parasite outcome

As expected, the proportion of mature larvae and pupae increased with chick age in both host species. We were able to confirm the findings of Kleindorfer et al. (2014) that increasing duration of chick survival corresponded to a higher pupation success of *P. downsi* larvae. In addition, there was a positive relationship between fly size and chick age, indicating the potentially higher reproductive success of flies that had more time to develop. However, the relationship between the proportion of mature larvae and pupae and chick age differed in the two hosts. In the warbler finch, the proportion of mature larvae and pupae was higher early in the nestling phase and increased more slowly; whereas in the small tree finch, it increased faster and reached higher levels in older nests. Kleindorfer et al. (2014) showed that *P. downsi* pupation success differed between the small ground finch and the small and medium tree finch but the factors causing the difference were unknown. In the closely related *Philornis torquans,* parasite reproductive success differed greatly between three host species (Manzoli et al. 2018) and depended on a complex interplay between parasite intensity, tolerance and resistance. In the present study, the higher proportion of mature larvae and pupae in warbler finch nests directly after hatching could be due to the higher infestation of incubating warbler finch nests compared to small tree finch nests. The finding that parasite intensity increases with chick age in both species, but that the proportion of mature larvae and pupae increased faster in the small tree finch than in the warbler finch might indicate that *P. downsi* larvae develop faster when parasitizing small tree finch chicks than warbler finch chicks. There are different possible (non-mutually exclusive) explanations for the differential increase in the proportion of mature larvae and pupae between the two hosts: (1) differences in the host-associated larval gut microbiome (Ben-Yosef et al. 2017), (2) differences in resistance due to differing immunocompetence, for example, the presence of *Philornis*-binding antibodies (Huber et al. 2010), and (3) difference in host blood quality due to differences in host diet. The warbler finch is insectivorous while the small tree finch is omnivorous (Filek et al. 2018) and thus, it is likely that their blood also differs in the ratio between carbohydrate versus nitrogen (the C:N ratio) ratio which is an indicator for food quality (reviewed in Aalto et al. 2015). These hypotheses could be tested by studying host and parasite microbiomes and measuring immunocompetence and blood quality (e.g., the C:N ratio) in relation to *P. downsi* parasitism. In addition to a more rapid increase in the proportion of mature larvae and pupae, small tree finch nests also yield a higher abundance of mature larvae and pupae at host resource termination because of the overall higher parasite intensity in these nests. Based on this body of evidence, it seems that the small tree finch is currently the better host for *P. downsi*.

### Change over time

We have witnessed a dynamic change in the relationship between the parasite and the two hosts over the last two decades: while parasite intensity increased in the small tree finch, it decreased in the warbler finch. One possible explanation for this pattern is the host specificity of *P. downsi* strains. Such host specificity is assumed to be based on genetic differences or differential patterns of expression (phenotypic acclimation) between parasite strains (reviewed in Little et al. 2006). If parasite fitness differs between *P. downsi* strains, parasite intensity could decrease or increase independently between hosts via differential survival of host-specific parasite strains. Under this scenario, each host-specific strain would be dependent on its host, which could potentially lead to stable host–parasite relationships between each host–parasite dyad (Papkou et al. 2016). However, genetic studies conducted to date indicate that *P. downsi* is a generalist, showing low genetic differentiation within the population in the Galápagos and that multiple mating and multiple nest infestations are common in this species (Dudaniec et al. 2008; Dudaniec et al. 2010). But it should be noted that these studies were conducted over ten years ago and did not focus on genetic variation between *P. downsi* attacking different hosts.

Another possible explanation for changes in parasite intensity over time are changes in host preference. The developmental time window to reach pupation is short and may have decreased over the last two decades (Kleindorfer et al. 2014). Currently, nestling failure is high and the average age of chick death is around 6 days in both hosts—the minimum developmental time window from egg-laying to pupation in *P. downsi*. Under such conditions, a parasite should prefer hosts that are conducive to fast development. In this scenario, host preference driven by competition between parasites for host resources would lead to the dynamic changes we currently observe. Multi-host systems do not necessarily lead to a stable equilibrium between the parasite and each host species. In the presence of reservoir hosts of differing vulnerability, parasite populations can continue to be at high levels in a robust host even if other, more vulnerable hosts are decreasing (reviewed in de Castro and Bolker 2005; Woolhouse et al. 2001). This could even lead to extinction of some host species (Fessl et al. 2017; Lafferty and Gerber 2002). The different outcomes of these two scenarios have differing implications with respect to the conservation of the unique land bird community of the Galápagos Islands. Hence it is crucial to understand the mechanisms and selective forces underlying this dynamic multi-host–parasite system, so that the most effective management strategies may be implemented. Host-specific parasite strains would call for specific management strategies for each host–parasite dyad, especially for rare species close to extinction like the mangrove finch (Fessl et al. 2010) or the medium tree finch (Dvorak et al. 2017; O’Connor et al. 2010b). In the second scenario, which does not predict a stable host–parasite equilibrium, *P. downsi* management should be twofold: first, the entire parasite population must be reduced and second, specific protection measures must be established for rare host species. Several studies have shown that Darwin’s finch chicks can be protected effectively by treating nests with the insecticide permethrin, which substantially reduces *P. downsi* numbers (Causton et al. 2019; Fessl et al. 2006a; Knutie et al. 2014; Knutie et al. 2016; Koop et al. 2013; Koop et al. 2011; O’Connor et al. 2014).

In summary, the present study provides new insights into the dynamics of the host–parasite interaction between *P. downsi* and two species of Darwin’s finches that may be useful in future efforts to design targeted *P. downsi* control measures in the Galápagos. We found differences in parasite intensity and prevalence between the two hosts and within the breeding cycle. During incubation, the parasitic fly infested warbler finch nests more frequently but once chicks hatched small tree finch nests contained more larvae, which could explain the higher virulence in this host. Additionally, long-term data indicate that infestation patterns of the small tree finch and the warbler finch have reversed during the last decade and results suggest faster larval development in small tree finch nests potentially leading to *P. downsi* preference for the better host.

## Electronic supplementary material

Below is the link to the electronic supplementary material.

Supplementary file1 (DOCX 24 KB)

## Data Availability

The datasets generated during and/or analyzed during the current study are available from the corresponding author on reasonable request.
