# Supplementary material for: Timing of infestation influences virulence and parasite success in a dynamic multi-host–parasite interaction between the invasive parasite, Philornis downsi, and Darwin’s finches
Source: Oecologia. 2020 Dec 1;195(1):249–59. doi: 10.1007/s00442-020-04807-5 (PMC7882474; doi:10.1007/s00442-020-04807-5)
Supplement: Supplementary file 1 — Supplementary file1 (DOCX 24 KB) [file 442_2020_4807_MOESM1_ESM.docx]

Supplemental Material for

**Timing of infestation influences virulence and parasite success in a dynamic multi-host parasite interaction between the invasive parasite, *Philornis downsi*, and Darwin’s finches**

Table S1: GLMs (binomial family and logit link function) testing for the effect of host species (small tree finch against warbler finch), standardized start of incubation and year on *Philornis downsi* prevalence in nests during the incubation and nestling phase. Intercept is the estimate for small tree finch nests in the year 2012.

|  | **Predictors** | **Estimates** | **95% CI** | **P** |
| --- | --- | --- | --- | --- |
| Incubation phase | (Intercept) | -0.99 | -2.87 to 0.79 | 0.28 |
|  | Speices Warbler finch | 1.94 | 0.97 to 3.05 | <0.001 |
|  | Start of incubation | 0.36 | -2.08 to 2.84 | 0.77 |
|  | Year 2014 | -1.88 | -3.63 to -0.36 | 0.02 |
|  | Year 2015 | -1.14 | -2.43 to 0.07 | 0.07 |
|  | Year 2016 | -1.66 | -3.01 to -0.41 | 0.01 |
|  | Year 2017 | -1.30 | -3.17 to 0.38 | 0.14 |
|  |  |  |  |  |
| Nestling phase | (Intercept) | 19.37 | -40.83 to 354.61 | 0.99 |
|  | Speices Warbler finch | 0.09 | -0.70 to 0.87 | 0.82 |
|  | Start of incubation | 0.22 | -1.26 to 1.75 | 0.77 |
|  | Year 2014 | -16.08 | -351.32 to 44.12 | 0.99 |
|  | Year 2015 | -17.01 | -352.25 to 43.19 | 0.99 |
|  | Year 2016 | -16.10 | -351.34 to 44.10 | 0.99 |
|  | Year 2017 | -17.86 | -353.10 to 42.34 | 0.99 |

Table S2: GLMs testing for the effect of host species (small tree finch against warbler finch), year and chick age on *Philornis downsi* intensity in nests during the incubation and nestling phase. Intercept is the estimate for small tree finch nests in the year 2012.

|  | **Predictors** | **Estimates** | **95% CI** | **P** |
| --- | --- | --- | --- | --- |
| Incubation phase | (Intercept) | 1.69 | 0.77 to 2.49 | <0.001 |
| (GLM, quasi-Poisson) | Species Warbler finch | -0.02 | -0.72 to 0.75 | 0.95 |
|  | Year 2014 | 0.25 | -0.94 to 1.30 | 0.66 |
|  | Year 2015 | -0.30 | -1.29 to 0.64 | 0.54 |
|  | Year 2016 | 0.01 | -0.87 to 0.89 | 0.98 |
|  | Year 2017 | 0.80 | -0.19 to 1.74 | 0.10 |
|  |  |  |  |  |
| Nestling phase | (Intercept) | 2.61 | 2.27 to 2.96 | <0.001 |
| (GLM, negative-binomial) | Species Warbler finch | -0.64 | -0.97 to -0.31 | <0.001 |
|  | Chick age (linear) | 0.20 | 0.12 to 0.28 | <0.001 |
|  | Chick age (quadratic) | -0.01 | -0.01 to -0.002 | 0.003 |
|  | Host species * chick age | 0.01 | -0.03 to 0.04 | 0.71 |
|  | Year 2014 | -0.09 | -0.31 to 0.12 | 0.40 |
|  | Year 2015 | 0.02 | -0.22 to 0.26 | 0.87 |
|  | Year 2016 | -0.05 | -0.31 to 0.22 | 0.72 |

Table S3: Comparison of chick age and *P. downsi* intensity, prevalence and proportion of mature larvae and pupae in small tree finch (STF) and warbler finch (WF) nests among years.

|  | **Year** | | | | | | | | | |
| --- | --- | --- | --- | --- | --- | --- | --- | --- | --- | --- |
|  | **2012** | | **2014** | | **2015** | | **2016** | | **2017** | |
|  | **STF** | **WF** | **STF** | **WF** | **STF** | **WF** | **STF** | **WF** | **STF** | **WF** |
| # nests with chicks | 37 | 46 | 73 | 96 | 54 | 49 | 29 | 36 | 36 | 72 |
| Parasite intensity^1^ | 37.4 ±4.19 | 26.2 ±2.90 | 30.7 ±2.62 | 24.6 ±1.63 | 39.1 ±3.72 | 22.6 ±2.50 | 35.9 ±4.54 | 25.0 ±3.08 | 30.6 ±4.03 | 17.5 ±2.07 |
| Parasite prevalence (%) | 100 | 100 | 96 | 98 | 93 | 94 | 100 | 94 | 83 | 85 |
| Age at end of nesting activity^1^ | 7.3 ±0.76 | 10.7 ±0.71 | 7.0 ±0.50 | 10.1 ±0.48 | 7.0 ±0.53 | 8.9 ±0.77 | 7.4 ±0.87 | 10.9 ±0.78 | 8.8 ±0.80 | 12.5 ±0.46 |
| Age at death^1^ | 5.4 ±0.54 | 6.0 ±0.74 | 6 ±0.43 | 6.0 ±0.45 | 6.2 ±0.47 | 6.5 ±0.75 | 6.5 ±0.80 | 7.4 ±1.09 | 5.9 ±0.81 | 6.2 ±0.98 |
| Prop. mature larvae^1^ | 0.46 ±0.06 | 0.56 ±0.04 | 0.58 ±0.04 | 0.69 ±0.03 | 0.44 ±0.05 | 0.62 ±0.05 | 0.51 ±0.07 | 0.56 ±0.06 | 0.64 ±0.06 | 0.77 ±0.03 |

^1^ Mean ±SE

Table S4: GLM (quasi-Poisson error structure) testing for the effect of host species (small tree finch against warbler finch) and year on chick age at the end of nesting activity (failure or fledged) and on chick age at death (failed nests only). Intercept is the estimate for small tree finch nests in the year 2012.

| **Response variable** | **Predictors** | **Estimates** | **95% CI** | **P** |
| --- | --- | --- | --- | --- |
| Chick age at end of nesting activity | (Intercept) | 2.01 | 1.89 to 2.13 | <0.001 |
|  | Species Warbler finch | 0.34 | 0.25 to 0.43 | <0.001 |
|  | Year 2014 | -0.05 | -0.18 to 0.08 | 0.44 |
|  | Year 2015 | -0.13 | -0.28 to 0.03 | 0.10 |
|  | Year 2016 | 0.02 | -0.15 to 0.18 | 0.84 |
|  | Year 2017 | 0.17 | 0.03 to 0.30 | 0.02 |
|  |  |  |  |  |
| Chick age at death | (Intercept) | 1.70 | 1.52 to 1.88 | <0.001 |
|  | Species Warbler finch | 0.05 | -0.08 to 0.18 | 0.43 |
|  | Year 2014 | 0.07 | -0.13 to 0.27 | 0.51 |
|  | Year 2015 | 0.12 | -0.09 to 0.33 | 0.27 |
|  | Year 2016 | 0.20 | -0.03 to 0.44 | 0.09 |
|  | Year 2017 | 0.07 | -0.18 to 0.32 | 0.60 |

Table S5: GLM (Poisson distribution, total number of nests as offset variable) testing for the effect of host species and chick age on the probability to of nesting failure. Intercept is the estimate for small tree finch.

| **Predictors** | **Estimates** | **95% CI** | **P** |
| --- | --- | --- | --- |
| (Intercept) | -2.11 | -2.37 to -1.86 | <0.001 |
| Species Warbler Finch | -0.75 | -1.16 to -0.35 | <0.001 |
| Chick age | -0.11 | -0.15 to -0.08 | <0.001 |
| Species x chick age | 0.02 | -0.03 to 0.08 | 0.42 |

Table S6 GLMs testing for the effect of host species (small tree finch against warbler finch), year and chick age on abundance and proportion of mature larvae and pupae in nests with chicks. Intercept is the estimate for small tree finch nests in the year 2012.

| **Response variable** | **Predictors** | **Estimates** | **95% CI** | **P** |
| --- | --- | --- | --- | --- |
| Abundance mature larvae | (Intercept) | 3.03 | 2.79 to 3.25 | <0.001 |
| (GLM quasi-Poisson) | Species Warbler finch | -0.26 | -0.43 to -0.09 | 0.003 |
|  | Year 2014 | 0.05 | -0.20 to 0.31 | 0.69 |
|  | Year 2015 | -0.03 | -0.32 to 0.26 | 0.82 |
|  | Year 2016 | -0.04 | -0.36 to 0.28 | 0.83 |
|  | Year 2017 | -0.08 | -0.37 to 0.21 | 0.59 |
|  |  |  |  |  |
| Proportion mature larvae | (Intercept) | -2.79 | -3.34 to -2.26 | <0.001 |
| (GLM, quasi-binomial) | Species Warbler finch | 0.93 | 0.39 to 1.47 | 0.001 |
|  | Chick age (linear) | 0.38 | 0.26 to 0.51 | <0.001 |
|  | Chick age (quadratic) | -0.001 | -0.01 to 0.002 | 0.18 |
|  | Host species * chick age | -0.12 | -0.18 to -0.07 | <0.001 |
|  | Year 2014 | 0.70 | 0.42 to 0.99 | <0.001 |
|  | Year 2015 | 0.27 | -0.03 to 0.57 | 0.08 |
|  | Year 2016 | -0.02 | -0.35 to 0.32 | 0.91 |
|  | Year 2017 | 0.76 | 0.42 to 1.10 | <0.001 |

Table S7 GLMs testing for the effect of host species (small tree finch against warbler finch) and year on total intensity, proportion of mature larvae and pupae, and abundance of mature larvae and pupae and abundance of immature small and medium larvae in nests with chicks of 5 days or younger. Intercept is the estimate for small tree finch nests in the year 2012.

| **Response variable** | **Predictors** | **Estimates** | **95% CI** | **P** |
| --- | --- | --- | --- | --- |
| Parasite intensity | (Intercept) | 3.13 | 2.86 to 3.39 | <0.001 |
| (GLM, quasi-Poisson) | Species Warbler Finch | -6.64 | -0.92 to -0.37 | <0.001 |
|  | Year 2014 | -0.16 | -0.49 to 0.17 | 0.34 |
|  | Year 2015 | -0.003 | -0.36 to 0.35 | 0.98 |
|  | Year 2016 | -0.16 | -0.60 to 0.27 | 0.48 |
|  | Year 2017 | -0.13 | -0.58 to 0.30 | 0.56 |
|  |  |  |  |  |
| Prop. mature larvae and pupae | (Intercept) | -1.43 | -1.94 to -0.97 | <0.001 |
| (GLM, quasi-binomial) | Species Warbler Finch | 0.76 | 0.33 to 1.20 | 0.001 |
|  | Year 2014 | 0.42 | -0.14 to 1.01 | 0.15 |
|  | Year 2015 | -0.02 | -0.65 tp 0.63 | 0.96 |
|  | Year 2016 | -0.48 | -1.39 to 0.36 | 0.28 |
|  | Year 2017 | 0.64 | -0.08 to 1.37 | 0.08 |
|  |  |  |  |  |
| Abundance mature larvae and pupae | (Intercept) | 1.50 | 0.96 to 1.96 | <0.001 |
| (GLM, quasi-Poisson) | Species Warbler Finch | -0.09 | -0.51 to 0.32 | 0.67 |
|  | Year 2014 | 0.15 | -0.41 to 0.75 | 0.61 |
|  | Year 2015 | -0.05 | -0.70 tp 0.62 | 0.89 |
|  | Year 2016 | -0.58 | -0.59 to 0.29 | 0.22 |
|  | Year 2017 | 0.30 | -0.41 to 1.01 | 0.40 |
|  |  |  |  |  |
| Abundance small larvae | (Intercept) | 2.93 | 2.61 to 3.21 | <0.001 |
| (GLM, quasi-Poisson) | Species Warbler Finch | -0.88 | -1.21 to -0.56 | <0.001 |
|  | Year 2014 | -0.28 | -0.65 to 0.10 | 0.14 |
|  | Year 2015 | 0.01 | -0.38 to 0.40 | 0.96 |
|  | Year 2016 | -0.06 | -0.53 to 0.40 | 0.80 |
|  | Year 2017 | -0.31 | -0.84 to 0.19 | 0.24 |

Table S8 Linear mixed model (LMM, with nest provenience as random factor) testing for the effect of host species, age of chicks at failure or fledging and sex of fly on *P. downsi* adult flies’ eye distance. Intercept is the estimate for female flies of small tree finch nests.

| **Predictors** | **Estimates** | **95% CI** | **P** |
| --- | --- | --- | --- |
| (Intercept) | 2.23 | 2.13 to 2.33 | <0.001 |
| Species Warbler Finch | 0.01 | -0.07 to 0.09 | 0.84 |
| Chick age | 0.04 | 0.03 to 0.05 | <0.001 |
| Sex (male) | 0.10 | 0.07 to 0.13 | <0.001 |
